# Double Dislocation of the Interphalangeal Joints of the Little Finger

**DOI:** 10.7759/cureus.37939

**Published:** 2023-04-21

**Authors:** Mahmut K Ozsahin, Muhammed Y Afacan, Onder Aydingoz

**Affiliations:** 1 Department of Orthopaedics and Traumatology, İstanbul University-Cerrahpaşa, Cerrahpaşa Faculty of Medicine, Istanbul, TUR

**Keywords:** little finger fracture, fracture accompanying double dislocation, rehabilitation, closed reduction, stepladder deformity, little finger, dip joint, pip joint, double dislocation

## Abstract

High-energy traumas may lead to the dislocation of joints with or without fractures. However, simultaneous double dislocation of the proximal and distal interphalangeal joints (PIP and DIP) in fingers shows up rarely. Although it can be inferred as simultaneous dislocation occurring during the same trauma, consecutive events should be taken into consideration. A 29-year-old, right-hand dominant male patient presented to the emergency room with a left little finger deformity after being hit by a ball while playing football. Despite the inability to move the little afteruent to this hyperextension injury, there was mild swelling, ecchymosis, and pain with no trace of laceration or neurovascular injury. PIP and DIP joint dislocations of the left-hand little finger with distal phalanx proximal fracture were detected on the radiograph indicating a stepladder deformity. Closed reduction was achieved by longitudinal traction and applying pressure over the base of the dislocated digit. Afterward, an aluminum finger splint was applied to the little finger in the functional position to prevent further damage. Re-evaluation radiographs revealed a successful reduction of both joints. Immobilization via an aluminum finger splint was recommended for three weeks. Subsequently, range of motion exercises and rehabilitation were started. Three-month follow-up revealed an almost full range of motion in both PIP and DIP joints without stiffness and pain. Although double dislocation seems to present with more painful and swollen fingers than single dislocations, it can also present with mild pain and swelling, as in this case. The little finger is easily exposed to traumas due to the lack of surrounding tissue. Therefore, double dislocation is mostly seen in the little finger. This case report briefly illustrates a rare incidence of double dislocation involving both the PIP and DIP joints of the little finger. Normal range of motion of both joints was reached by early reduction followed by timely rehabilitation.

## Introduction

High-energy traumas may lead to the dislocation of joints with or without fractures. However, simultaneous double dislocation of the proximal and distal interphalangeal joints (PIP and DIP) in fingers shows up rarely. When the tip of the little finger encounters trauma in a hyperextended position, the dorsally directed force ruptures the volar capsule of the involved finger’s interphalangeal joints leading to the displacement of the distal phalanx base to the dorsum of the middle phalanx tip. The force mechanism continues, and the middle finger moves dorsally to the proximal phalanx resulting in double dislocations, which is also termed stepladder deformity in the literature [1,2]. Although it can be inferred as simultaneous dislocation occurring during the same trauma, these consecutive events should be taken into consideration. As a result of the trauma mechanism, the double dislocations mentioned in the literature were dorsally displaced, and there is no case showing double volar dislocation.

## Case presentation

**Figure 1:**
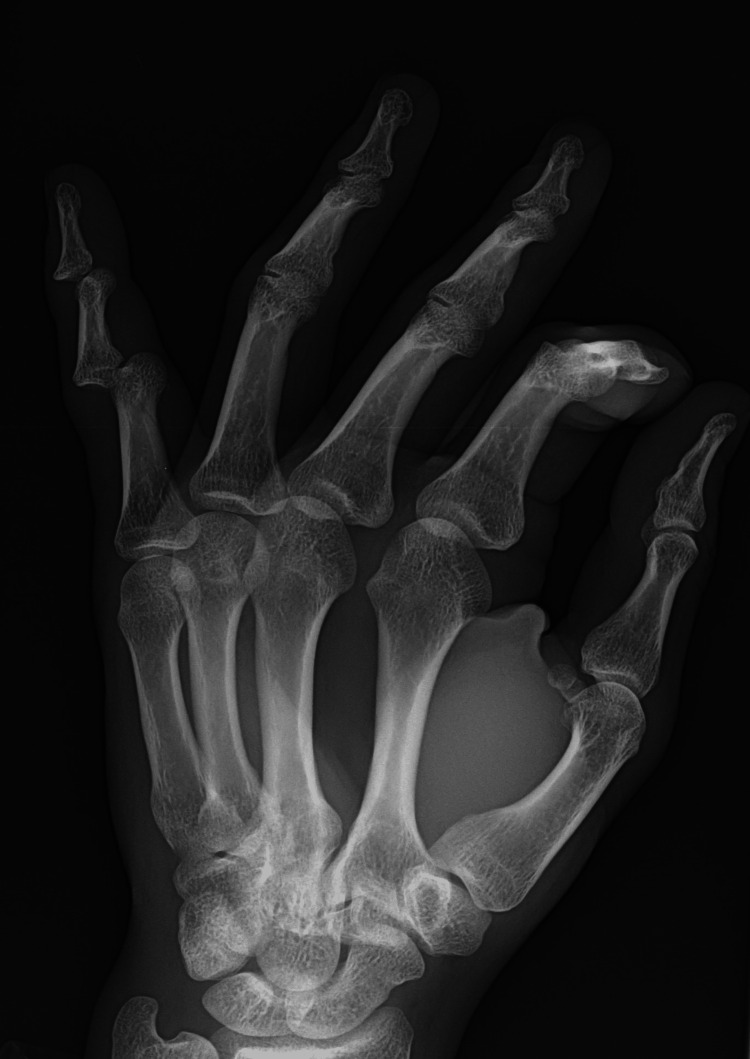
Oblique left-hand radiograph. Oblique radiograph of the patient’s left hand showing double dislocation of both proximal and distal interphalangeal joints of the little finger.

**Figure 2:**
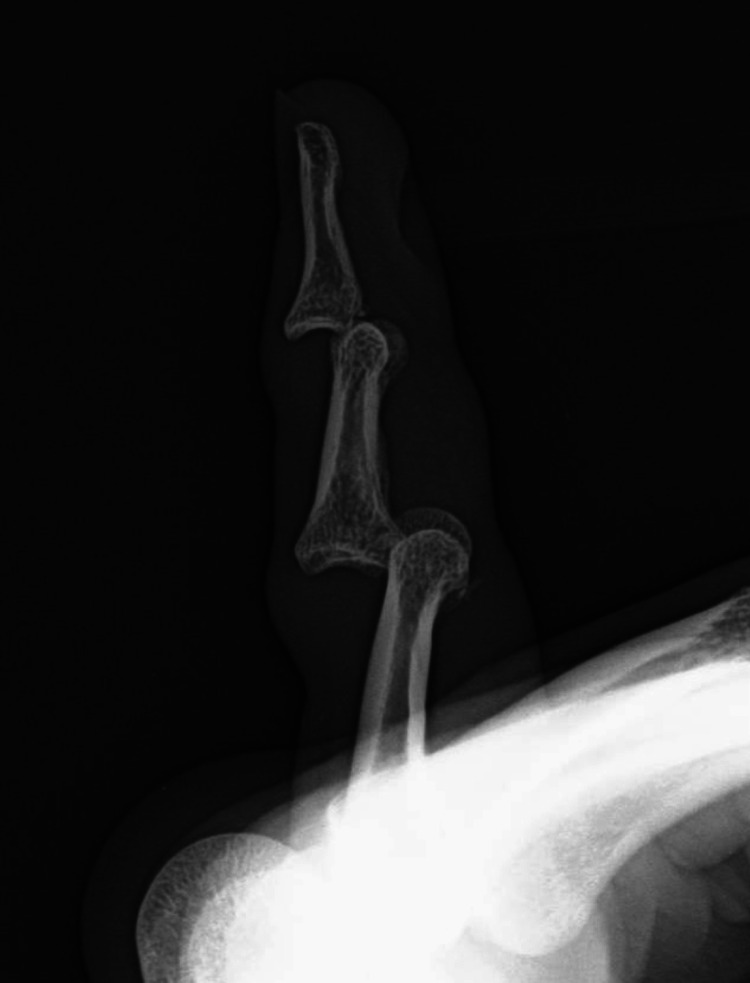
Lateral left little finger radiograph before reduction. Lateral radiograph of the patient’s left little finger showing double dislocation of both proximal and distal interphalangeal joints.

A 29-year-old, right-hand dominant male patient with hypereosinophilic syndrome and hypertension who regularly used cilostazol, warfarin, carbamazepine, amlodipine, and pegylated interferon alpha-2a presented to the emergency room with left little finger deformity after being hit by a ball while playing football. Despite the inability to move the little finger subsequent to the hyperextension injury, there was mild swelling, ecchymosis, and pain with no trace of laceration or neurovascular injury. PIP and DIP dislocations of the left-hand little finger with distal phalanx proximal fracture were detected on the radiograph, indicating a stepladder deformity (Figures 1, 2).

**Figure 3:**
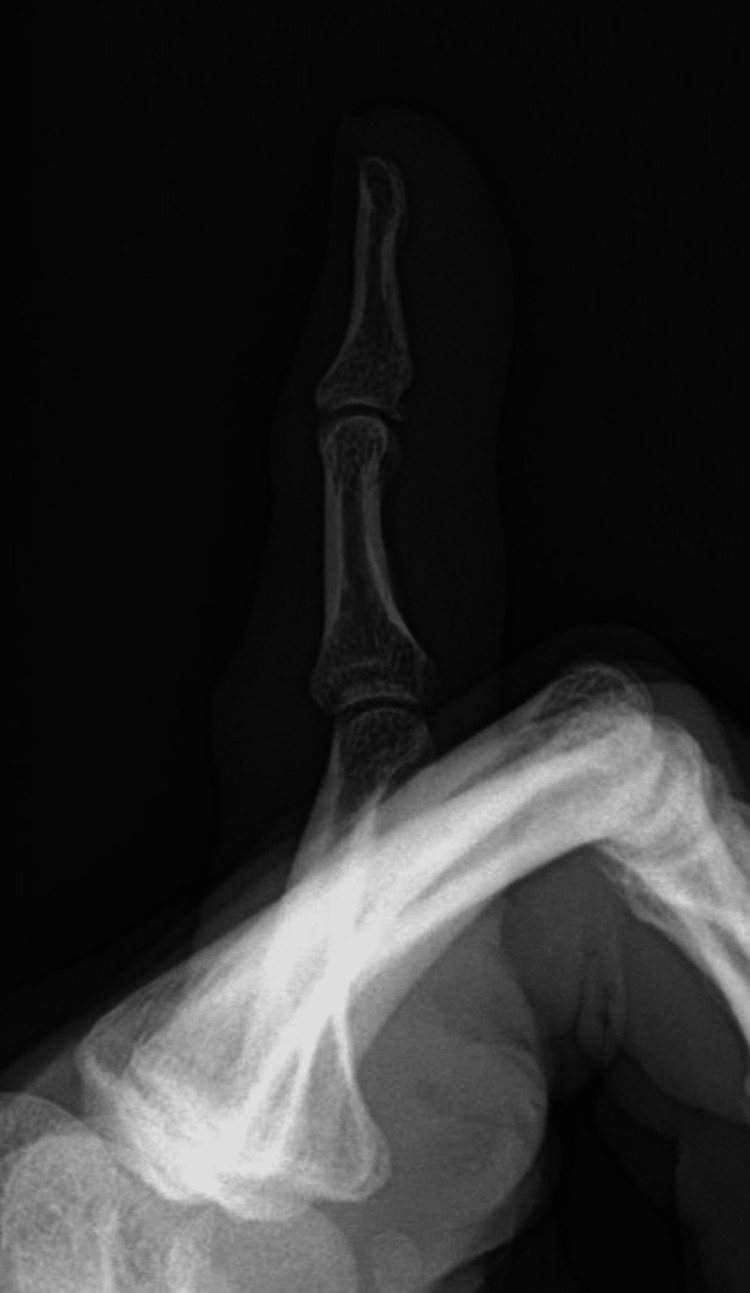
Lateral left little finger radiograph after reduction. Lateral radiograph of the patient’s left little finger after reduction showing the proper alignment of both joints.

Closed reduction was achieved by longitudinal traction and applying pressure over the base of the dislocated digit. Afterward, an aluminum finger splint was applied to the little finger in the functional position at 45 degrees of metacarpophalangeal flexion and 15 degrees of both PIP and DIP joints’ flexion to prevent further damage. Re-evaluation radiographs revealed a successful reduction of both joints (Figure 3).

Immobilization via an aluminum finger splint was recommended for three weeks. Subsequently, range of motion exercises and rehabilitation were started. Six-month follow-up revealed full functional recovery with a full range of motion in both PIP and DIP joints without stiffness and pain.

## Discussion

Although double dislocation seems to present with more painful and swollen fingers than single dislocation, it can also present with mild pain and swelling, as in this case. The little finger is easily exposed to traumas due to the lack of surrounding tissue. Therefore, double dislocation is mostly seen in the little finger. Despite the little finger predominance, there are cases in the literature involving the thumb with double dislocation of the metacarpophalangeal and interphalangeal joints [2,3]. Although the reduction was achieved easily in the presented case, there are also reports of irreducible cases [4]. In the case of Hara et al., the DIP joint remained unstable after reduction maneuvers. Moreover, a closed reduction of the PIP joint could not be achieved. Subsequent MRI revealed ulnar transposition of the flexor digitorum profundus tendon at the PIP joint level. Then, open reduction and fixation of the PIP joint was performed, after which the DIP joint became more stable. Ransbeeck et al. reviewed all cases of double dislocations of the fingers in the literature. They reported a male and right-hand predominance, with most cases caused by sports trauma. According to their study, the majority of double dislocations occur in the little finger followed by the ring, middle and index fingers, and the thumb [1]. Our case resembles the results of the review regarding the injury mechanism, gender, and involved finger, whereas the hand side does not. Raval et al. elaborated on sports accidents and emphasized football as the most common sport. They also defined falls as the second most common accident type [2]. According to Kim et al., the timing of closed reduction plays a vital role in determining the success of the treatment. The derangement of the hyaline cartilage and synovial tissue will lead to degenerative changes if the early reduction is delayed. The delay in reduction may cause loss of normal range of motion of the interphalangeal joints. Therefore, one week of delay in closed reduction leads to open reduction [3].

## Conclusions

This case report briefly illustrated a rare incidence of double dislocation involving both the PIP and DIP joints of the little finger. The normal range of motion of both joints was achieved. Although the double dislocation of the little finger was accompanied by fractures of the proximal ends of the middle and distal phalanges, the proper alignment and full range of motion were obtained without any surgical intervention due to the early reduction followed by timely rehabilitation. The patient’s history of hypereosinophilic syndrome should be taken into special consideration in further investigations to determine whether this disease causes ligament and joint laxity.
